# CoCl_2_ simulated hypoxia induce cell proliferation and alter the expression pattern of hypoxia associated genes involved in angiogenesis and apoptosis

**DOI:** 10.1186/s40659-019-0221-z

**Published:** 2019-03-15

**Authors:** Nishant Kumar Rana, Priya Singh, Biplob Koch

**Affiliations:** Department of Zoology, Institute of Science, Banaras Hindu University, Varanasi, U. P. 221005 India

**Keywords:** Hypoxia, Apoptosis, CoCl_2_, HIF-1α, VEGF, p53, BAX

## Abstract

**Background/aims:**

Hypoxia microenvironment plays a crucial role during tumor progression and it tends to exhibit poor prognosis and make resistant to various conventional therapies. HIF-1α acts as an important transcriptional regulator directly or indirectly associated with genes involved in cell proliferation, angiogenesis, apoptosis and energy metabolism during tumor progression in hypoxic microenvironment. This study was aimed to investigate the expression pattern of the hypoxia associated genes and their association during breast cancer progression under hypoxic microenvironment in breast cancer cells.

**Methods:**

Cell proliferation in MCF-7 and MDA-MB-231 cell lines treated with different concentration of CoCl_2_ was analyzed by MTT assay. Flow cytometry was performed to check cell cycle distribution, whereas cell morphology was examined by phase contrast microscopy in both the cells during hypoxia induction. Expression of hypoxia associated genes HIF-1α, VEGF, p53 and BAX were determined by semiquantitative RT-PCR and real-time PCR. Western blotting was performed to detect the expression at protein level.

**Results:**

Our study revealed that cell proliferation in CoCl_2_ treated breast cancer cells were concentration dependent and varies with different cell types, further increase in CoCl_2_ concentration leads to apoptotic cell death. Further, accumulation of p53 protein in response to hypoxia as compare to normoxia showed that induction of p53 in breast cancer cells is HIF-1α dependent. HIF-1α dependent BAX expression during hypoxia revealed that after certain extent of hypoxia induction, over expression of BAX conquers the effect of anti-apoptotic proteins and ultimately leads to apoptosis in breast cancer cells.

**Conclusion:**

In conclusion our results clearly indicate that CoCl_2_ simulated hypoxia induce the accumulation of HIF-1α protein and alter the expression of hypoxia associated genes involved in angiogenesis and apoptosis.

**Electronic supplementary material:**

The online version of this article (10.1186/s40659-019-0221-z) contains supplementary material, which is available to authorized users.

## Background

Breast cancer is the most commonly diagnosed cancer in women. About one out of eight women develop breast cancer throughout life [1]. Early detection through screening programs and new therapeutic strategies have improved the chances to survive; however, many women still die because of metastasis. Prognosis and survival rates for breast cancer vary according to cancer type, stage, treatment, and geographical location of the patient. Survival rates in western world are quite high as compare to developing countries and more than 8 out of 10 women diagnosed with breast cancer survive for at least 5 years in England (84%). Whereas in India incidence of breast cancer is rapidly rising but the survival rate is not even more than 60% [2]. Hypoxia can be defined as the reduction of oxygen or increase in consumption of oxygen relative to the supply in cells, tissue or organs. It is well known that hypoxia is associated with poor prognosis [3], increased angiogenesis [4], tumor growth and resistance to several therapies [5]. Although hypoxia is toxic to both cancer cells and normal cells, cancer cells undergo genetic and adaptive changes that allow them to survive and even proliferate in a hypoxic environment [6, 7]. Multiple studies suggest that hypoxia inducible factor alpha (HIF-1α) get stabilized during hypoxic condition and regulates various genes involved in angiogenesis or apoptosis. It was reported that HIF-1α, VEGF (vascular endothelial growth factor) and p53 play an important role in radiation resistance of tumor cells therefore they can be the potential therapeutic targets to eradicate cancer [8–10]. Hypoxia has been described as p53 inducer and as we know p53 plays important role in various pathways of cell cycle delay, apoptosis and cells survival in hypoxic microenvironment [11]. Due to increase in expression of anti-apoptotic proteins cancer cells became resistant to chemotherapy and radiotherapy. Whereas reports suggest that BAX gene surmount the effect of anti-apoptotic proteins and over expression of BAX gene can lead to apoptosis in cancer cells [12–14]. However molecular mechanism responsible for the hypoxic survival of breast cancer cells are not well characterised therefore the direct interaction among HIF-1α, p53 and BAX may affect hypoxia induced apoptosis.

Therefore, the present study was undertaken to established a relation between CoCl_2_ simulated cell proliferation and apoptosis in breast cancer cells under hypoxic condition and to investigate the expression pattern of these factors and their association during breast cancer progression under hypoxic microenvironment.

## Materials and methods

### Cell culture

Two human breast cancer cell lines (MCF-7 and MDA-MB-231) were grown in Dulbecco Modified Eagle Medium (DMEM; Gibco, Invitrogen, Carlsbad, CA, USA) supplemented with 10% fetal bovine serum (FBS; Gibco, Invitrogen, Carlsbad, CA, USA), 100 units/mL penicillin and 100 µg/mL streptomycin (Cellclone; Genetix Biotech Asia Pvt. Ltd.) in a CO_2_ incubator (Heal Force HF 90 Shanghai China) with humidified air containing 5% CO_2_ at 37 °C. 1 × 10^6^ cells from each cell lines (MCF-7 and MDA-MB-231) were seeded in T-25 culture flask (Eppendorf, Hamburg, Germany) and left for 24 h in CO_2_ incubator.

### Preparation of CoCl_2_ stock solution and hypoxia treatment

Stock solution 25 mM of cobalt chloride (CoCl_2_) was prepared in sterile distilled water and further diluted in medium in order to obtain the final desired concentrations. MCF-7 as well as MDA-MB-231 cells were cultured in DMEM supplemented with 10% FBS and 100 units/mL penicillin and 100 µg/mL streptomycin in standard CO_2_ incubator. After 24 h of incubation, the breast cancer cell lines were treated with different concentration of CoCl_2_.

### MTT cell proliferation assay

Briefly, 4000 cells from each cell line (MCF-7 and MDA-MB-231) were seeded treated in 96-well plate with increasing concentration of CoCl_2_ (0–250 µM for MCF-7 and 0–200 µM for MDA-MB-231) to induce hypoxia. The cells were then incubated for 72 h in a CO_2_ incubator with humidified atmosphere at 37 °C and 5% CO_2_. The media was replaced and 10 µL of MTT (Himedia, India) from the stock (5 mg/mL in PBS) was added into each well of 96-well plate and incubated for 2 h at 37 °C. MTT was removed and 100 µL DMSO was added to dissolve the formazan crystal and left for 30 min at 37 °C. Absorbance was detected in ELISA plate reader at 570 nm.

### Cell cycle analysis by flow cytometry

Distribution of cells in cell cycle was analysed by flow cytometry. In brief, MCF-7 and MDA-MB-231 cells were seeded in a 6-well cell culture plate and treated with different concentration of CoCl_2_ to induce hypoxia (50, 100, 150 and 200 µM for MCF-7; 10, 20, 25 and 50 µM for MDA-MB-231 cells). The cell harvested followed by fixation in 70% chilled ethanol. Staining of the cells was carried out with PI-RNase solution (1 mg/mL PI, 0.1% V/V Triton X-100 and 10 mg/mL RNase) and analysed in FACScan using Cell Quest software (Becton–Dickinson).

### Study of cell morphology during hypoxia induction

Cells were incubated at a density of 4000 cells per well onto a 96-well cell culture plate for 72 h in a standard CO_2_ incubator and images were taken in inverted phase contrast microscope after every 24 h following treatment with different concentration of CoCl_2_ to observe the morphology.

### RT-PCR

Following treatment of CoCl_2_ in MCF-7 and MDA-MB-231, RNA were isolated using TRI reagent (Sigma-Aldrich) according to manufacturer’s instruction. Consequently c-DNA was synthesised with 1 µg RNA by RevertAid H Minus First Strand c-DNA synthesis kit (Thermo Fisher Scientific) according to the manufacturer protocol. Further to check differential expression pattern of HIF-1α, VEGF, p53 and BAX; amplification of these genes were performed in a DNA Thermal Cycler (Applied Biosystem) with gene specific primers obtained from Eurofins Genomics India Pvt. Ltd. (Additional file 1: Table S1), while reaction procedure during PCR amplification has been used as follows: β-actin: 28 cycles at 95 °C for 4 m, 95 °C for 45 s, 50 °C for 45 s, 72 °C for 45 s; HIF-1α: 28 cycles at 95 °C for 4 m, 95 °C for 45 s, 51 °C for 45 s, 72 °C for 45 s; VEGF: 35 cycles at 95 °C for 4 m, 95 °C for 45 s, 57.3 °C for 45 s, 72 °C for 45 s; p53: 32 cycle at 95 °C for 4 m, 95 °C for 45 s, 56.5 °C for 45 s, 72 °C for 45 s; BAX: 32 cycles at 95 °C for 4 m, 95 °C for 45 s, 54.5 °C for 45 s, 72 °C for 45 s; final extension at 72 °C for 10 min. In order to evaluated the differential expression pattern of HIF-1α, VEGF, p53 and BAX with increasing concentration of CoCl_2_ relative densitometry values were calculated after normalisation with β-actin.

### Western blot analysis

MCF-7 and MDA-MB-231 were treated with selected concentration of CoCl_2_ (50 µM, 100 µM, 150 µM for MCF-7 and 10 µM, 25 µM, 50 µM and 100 µM for MDA-MB-231) and incubated under CO_2_ incubator for 24 h. Total proteins were extracted by RIPA buffer (Bio-Chemax, Axiva Sichem Biotech, India) and protein concentration were determined by standard bicinchoninic acid (BCA; Bio vision USA) method [15]. 40 µg of protein was loaded into each well and separated by sodium dodecyl sulphate-polyacrylamide gel electrophoresis (SDS-PAGE) followed by transfer to polyvinylidene fluoride (PVDF; Merck Millipore, USA). Consequently, primary antibody for each protein like anti-β-actin (Mouse monoclonal antibody obtained from ambion by life technologies; Cat. No. AM4302), anti-p53 (Mouse monoclonal antibody from Puregene by Genetix Biotech Asia Pvt. Ltd; Cat. No. GX-8701M), anti-HIF-1α (Rabbit monoclonal antibody by Invitrogen Cat. No. 700505) and anti-VEGF (Rabbit polyclonal antibody from Puregene by Genetix Biotech Asia Pvt. Ltd) was added in blocking solution individually with standardized dilution (1:500 to 1:1000) and incubated for 16 h at 4 °C. The membrane was washed three times in TBST and then incubated for 2 h with secondary antibody (for β-actin and p53 Rabbit anti-mouse IgG-ALP from GeNei Cat. No. 621100980011730, while for HIF-1α and VEGF Goat anti-rabbit IgG-ALP has been used from GeNei Cat. No. 621100180011730) (secondary antibodies have been used with 1:16,000 dilution). Subsequently membrane was washed thrice in TBST and exposed to the NBT/BCIP (AMRESCO). The expression of protein was detected and captured in E-Gel Imager (Thermo Fisher, USA).

### Real-time PCR (qPCR)

qPCR has been performed by using following protocol: denaturation program (95 °C for 10 min), amplification and quantification program repeated 40 times (95 °C for 15 s, 60 °C for 1 min, 95 °C for 30 s with a single fluorescence measurement), melting curve program (60–95 °C with a heating rate of 0.1 °C per second and a continuous fluorescence measurement) and finally a cooling step to 4 °C.

### Statistical analysis

All values obtained were represented as mean ± standard error (SE) of at least three independent experiments. Statistical analysis were performed with SPSS version 16.0 (SPSS, Chicago, IL, USA). Differences between control (normoxia, N) and CoCl_2_ treated groups (hypoxia) were evaluated by One-way ANOVA followed by Tukey post hoc test. The P value less than 0.05 was considered as statistically significant.

## Results

### MTT assay

Both the cell lines showed significant proliferation in CoCl_2_ treated cells as compare to untreated cells, but the concentration of CoCl_2_ that showed maximum proliferation differ in two cell lines. MCF-7 cell line showed maximum proliferation (P < 0.05) when treated with 150 µM of CoCl_2_, whereas MDA-MB-231 promotes significant increased in proliferation (P < 0.05) at 25 µM concentration. However, it worth to mention that further increased in the CoCl_2_ concentration gradually decrease their proliferation rates in both the cell line which showed their inhibitory effect after certain concentration.

### Cell cycle distribution analysis by FACS

**Fig. 1:**
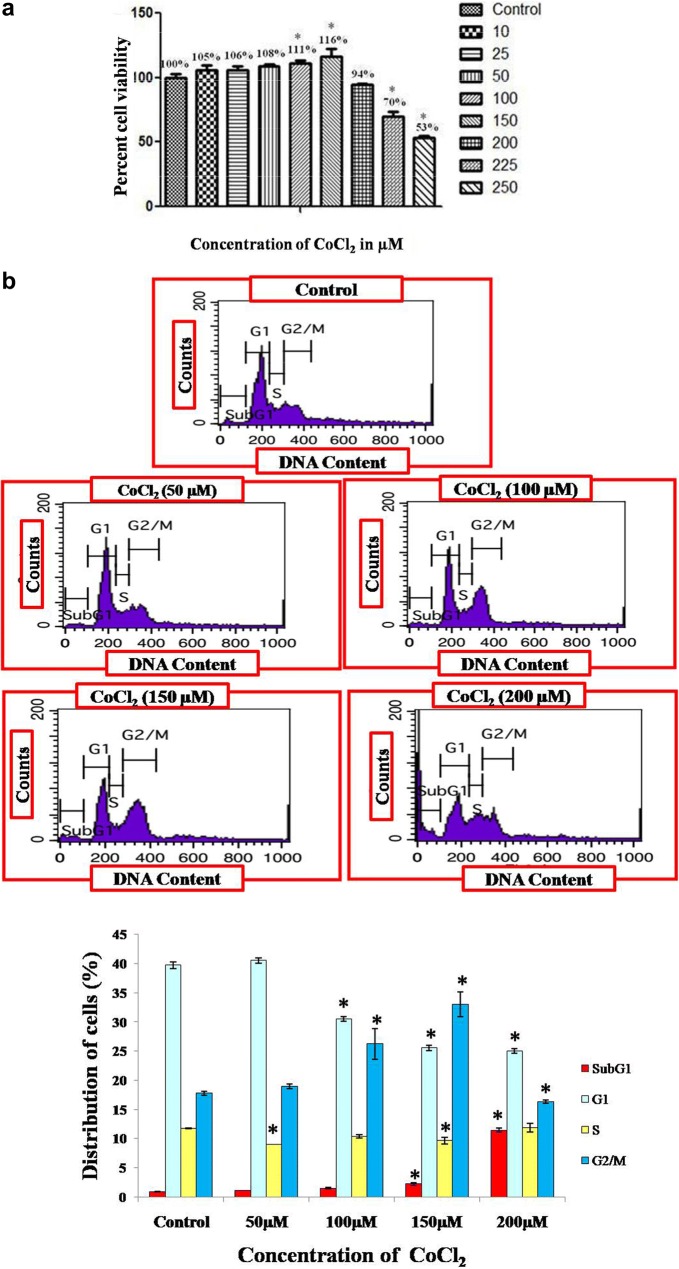
Cell proliferation in MCF-7 cells after 72 hrs treatment with different concentration of CoCl_2_ was determined by MTT assay and represented in bar diagram (**a**), whereas **b** illustrates graphical represenation of flow cytometry data for cell cycle distribution of MCF-7 cells exposed to CoCl_2_ in concentration dependent manner obtained from histogram analysis through FACScan (Becton Dickinson). *Denotes significant difference (P <0.05) as compared to control

**Fig. 2:**
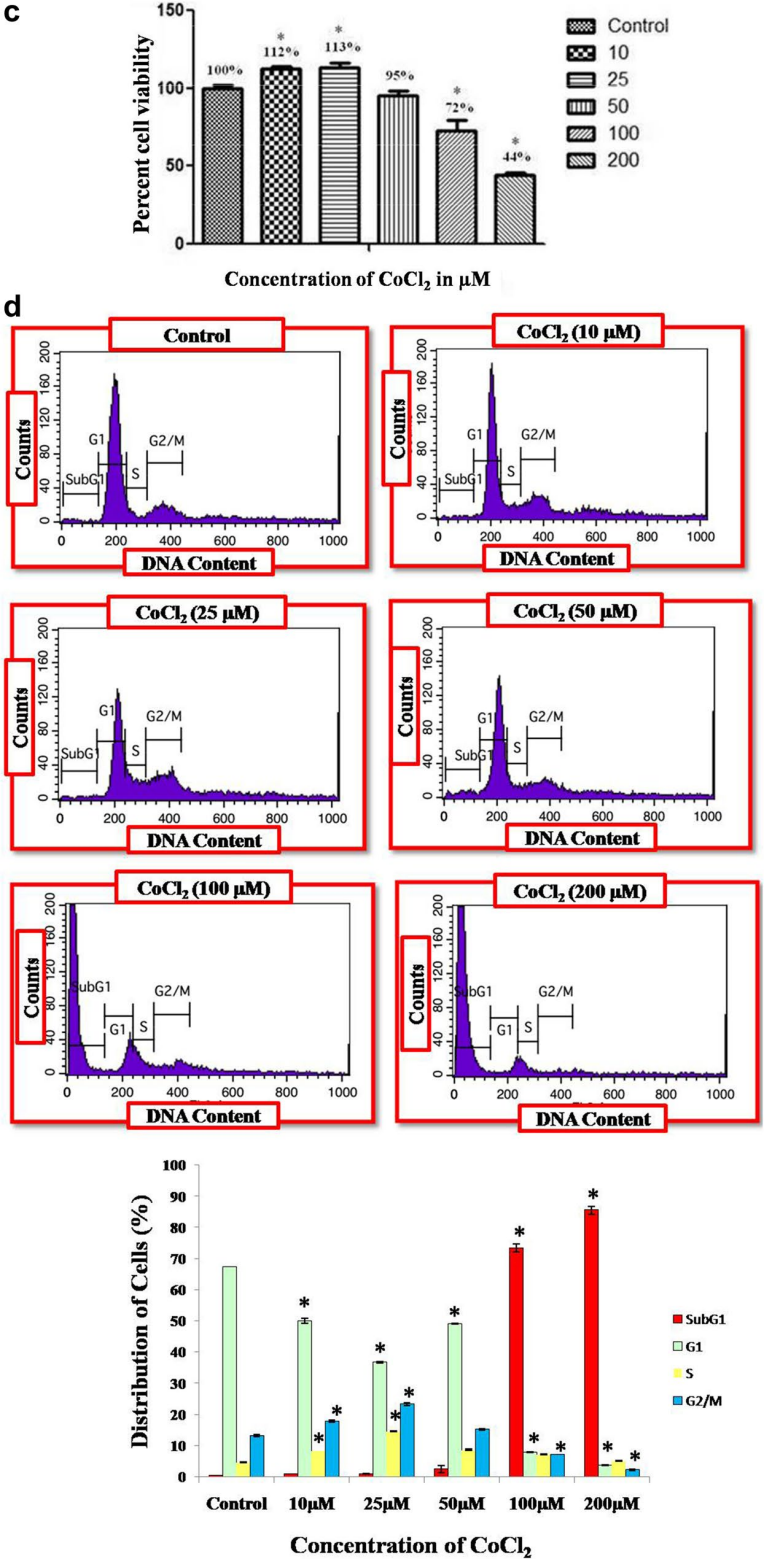
Cell proliferation in MDA-MB-231 cells after 72 hrs treatment with different concentration of CoCl_2_ was determined by MTT assay and represented in bar diagram (**a**), whereas **b** illustrates graphical represenation of flow cytometry data for cell cycle distribution of MCF-7 cells exposed to CoCl_2_ in concentration dependent manner obtained from histogram analysis through FACScan (Becton Dickinson). *Denotes significant difference (P <0.05) as compared to control

CoCl_2_ treated cells showed significant increase in cell population particularly at G2/M phase in both the cell lines after 72 h of treatment as compare to untreated cells. The increase in G2/M phase was dose dependent of maximum increased was observed when CoCl_2_ was given 150 µM in MCF-7 cell and 25 µM in case of MDA-MB-231 cells. Further increase in concentration of CoCl_2_ leads to enhancement of Sub G1 cell population also in both the cell lines. Our results also depicts that during hypoxia induction by CoCl_2_ in MDA-MB-231cells as the concentration increases up to 25 µM cell population in S phase also increases significantly in dose dependent manner (Figs. 1, 2).

### Effects of CoCl_2_ induced hypoxia on cells morphology

**Fig. 3:**
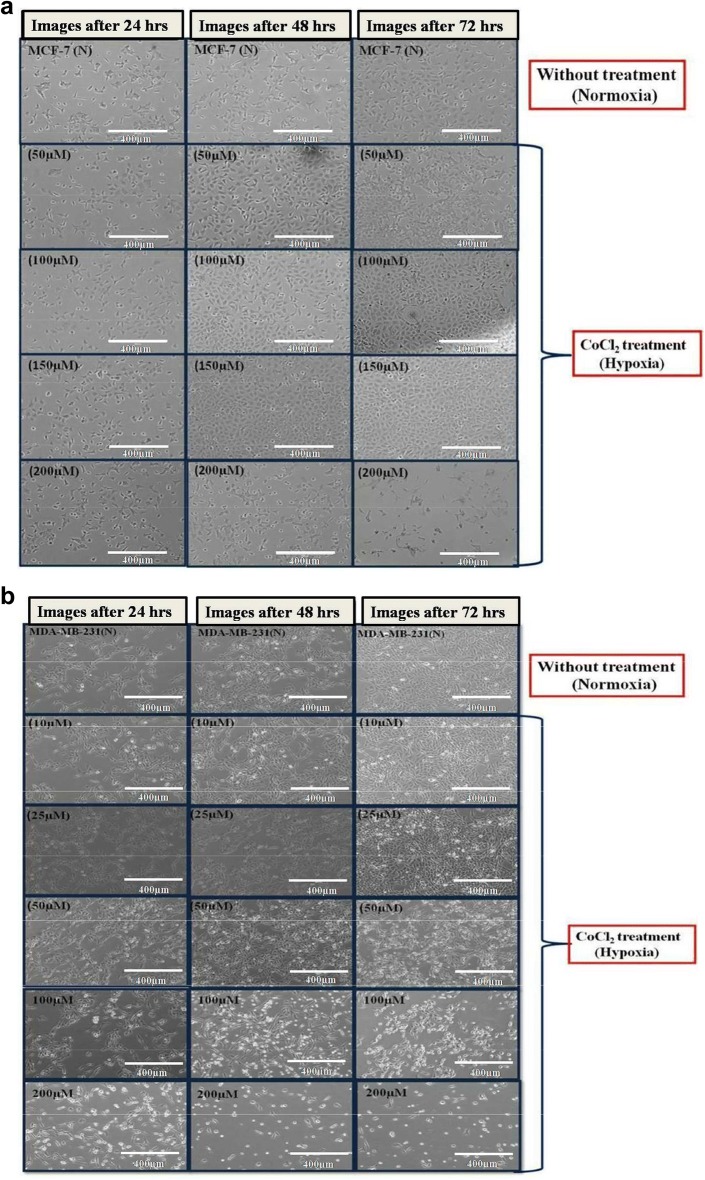
Cell proliferation with increase in concentration of CoCl_2_ (MCF-7 50–200 µM; MDA-MB-231 10–200 µM) in MCF-7 (**a**) and MDA-MB-231 (**b**) cell lines were analysed under phase contrast microscopy in a time dependent manner. Maximum cell proliferation has been observed at 150 µM and 25 µM in MCF-7 and MDA-MB-231 respectively. Cell morphology was also analysed at ×10 objective in inverted light microscope (EVOS FL cell imaging system, Life Technologies, USA) where cytoplasmic condensation and cell shrinkage were observed at higher CoCl_2_ concentration i.e., above 150 µM in case of MCF-7 (**a**) and above 25 µM in MDA-MB-231 (**b**) which ultimately leads to cell death

The effect of CoCl_2_ in cell proliferation has also been observed in a dose dependent manner under an inverted light microscope for both the cell lines (MCF-7 and MDA-MB-231) treated with different concentration of CoCl_2_. It was observed that there was an increased in the cell population in a dose dependent manner up to a certain concentration (50–150 µM in MCF-7 and 10–25 µM in MDA-MB-231) whereas further increase in concentration of CoCl_2_ exhibit cell shrinkage and cytoplasmic condensation which ultimately leads to cell death [16, 17]. Cell death at higher concentration of CoCl_2_ in beyond its proliferative dose (150 µM for MCF-7; 25 µM for MDA-MB-231) clearly indicates that the toxicity of compound (Fig. 3a, b).

### Differential expression of hypoxia associated genes

PCR results show a band of expected size for each gene (395 bp for β-actin; HIF-1α-252 bp; VEGF-189 bp; p53-173 bp; BAX-256 bp), which reflects the differential expression of hypoxia associated genes in both cells before and after CoCl_2_ treatment. Analysis of the observed bands clearly indicate the degree of expression of HIF-1α and VEGF increases in a dose dependent manner in both the cell lines (50–150 µM in MCF-7; 10–25 µM in MDA-MB-231) which strongly support the hypoxia induction by CoCl_2_ [18–20]. Increase in p53 expression in a dose dependent manner was also observed in breast cancer cells treated with CoCl_2_ which depicts that hypoxia induces p53 expression from its basal level as compared to cells grown under normoxia [11]. It was also observed that expression of pro-apoptotic genes like BAX [12] increases in a dose dependent manner and a significant increase in expression of BAX has been observed at 25 µM. Further increase in CoCl_2_ concentration enhance the extent of hypoxia which leads apoptotic cell death.

### Western blot for analysis of expression of HIF-1α, VEGF and p53 protein

Further, in order to investigate CoCl_2_ simulated hypoxia induction in both the cell lines (MCF-7 and MDA-MB-231) expression of HIF-1α and VEGF protein were detected by western blot where β-actin is used as internal control. As hypoxia accumulates p53 protein [21] therefore expression level of p53 protein was also investigated in one of these two cell lines (MDA-MB-231) to confirmed the role of p53 in cell survival during hypoxic stress. A band of an expected size (β-actin— ~ 42 kDa; HIF-1α— ~ 92 kDa; VEGF— ~ 45 kDa; p53— ~ 53 kDa) for each protein were obtained with their specific primary antibodies. Interestingly, a dose dependent (In MCF-7: 50–150 µM; MDA-MB-231: 10–25 µM) increase in HIF-1α and VEGF protein expression has been observed in both the cell lines which strongly supports the hypoxia induction by CoCl_2_. In contrast to this p53 protein expression was also increased in a dose dependent manner as hypoxia has been considered as p53 inducer in cancer cells.

### Real-time PCR

**Fig. 4:**
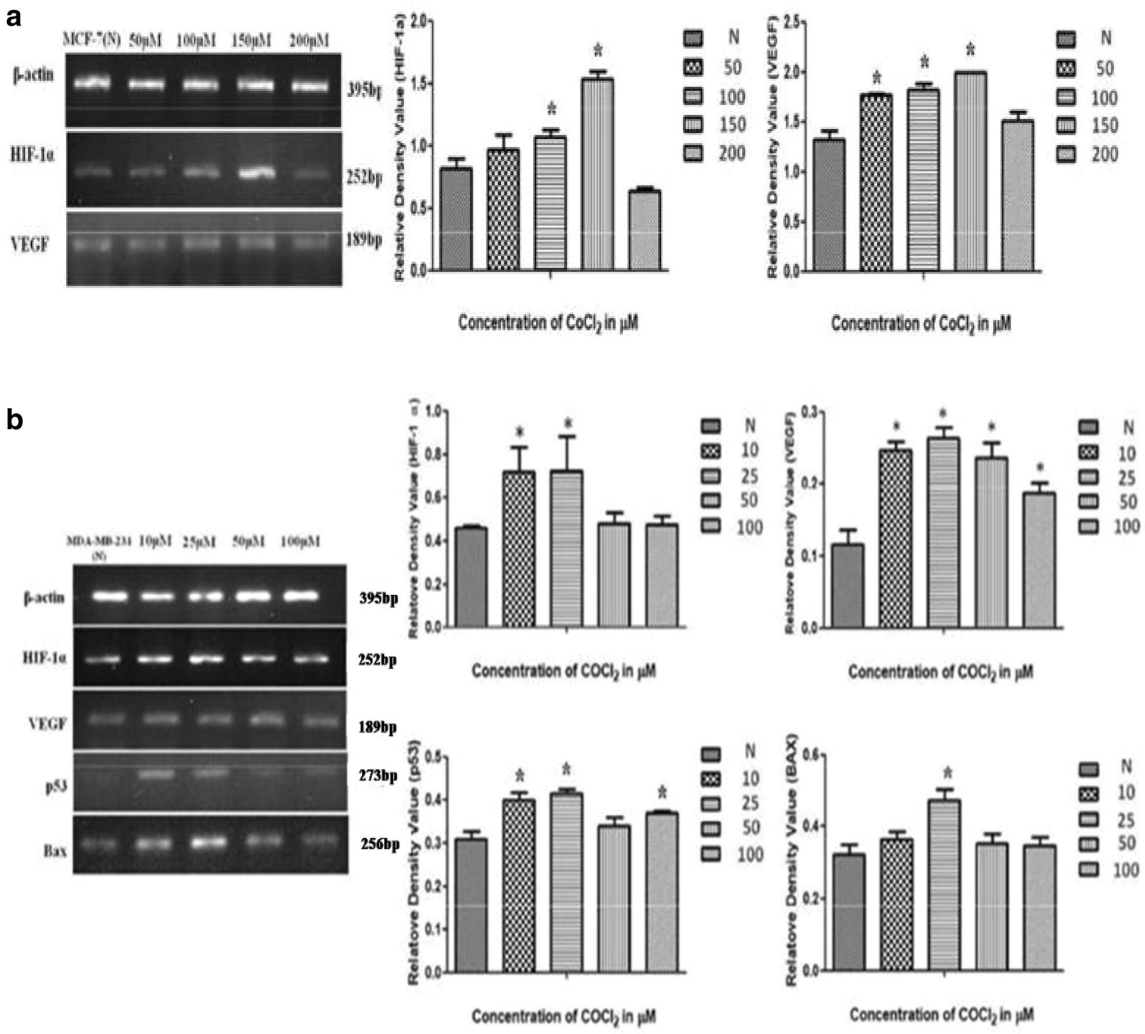
Differential expression pattern of HIF-1α and hypoxia associated genes involved in angiogenesis and apoptosis like VEGF, p53 and BAX in breast cancer cells, where graphical data represents the relative density value after densitometry analysis of band of PCR amplified product. **a** Depicts the expression pattern of β-actin, HIF-1α and VEGF in MCF-7 cells treated with different concentration of CoCl_2_ as compare to control by RT-PCR, similarly **b** shows expression of β-actin, HIF-1α, VEGF, p53 and BAX genes in MDA-MB-231 cells treated with increasing concentration of CoCl_2_, where it was clearly observed that expression of these genes increases significantly in a dose dependent manner. Error bar represents mean ± SEM of minimum three independent experiments and * Denotes significant difference (P < 0.05) as compared to control (N)

**Fig. 5:**
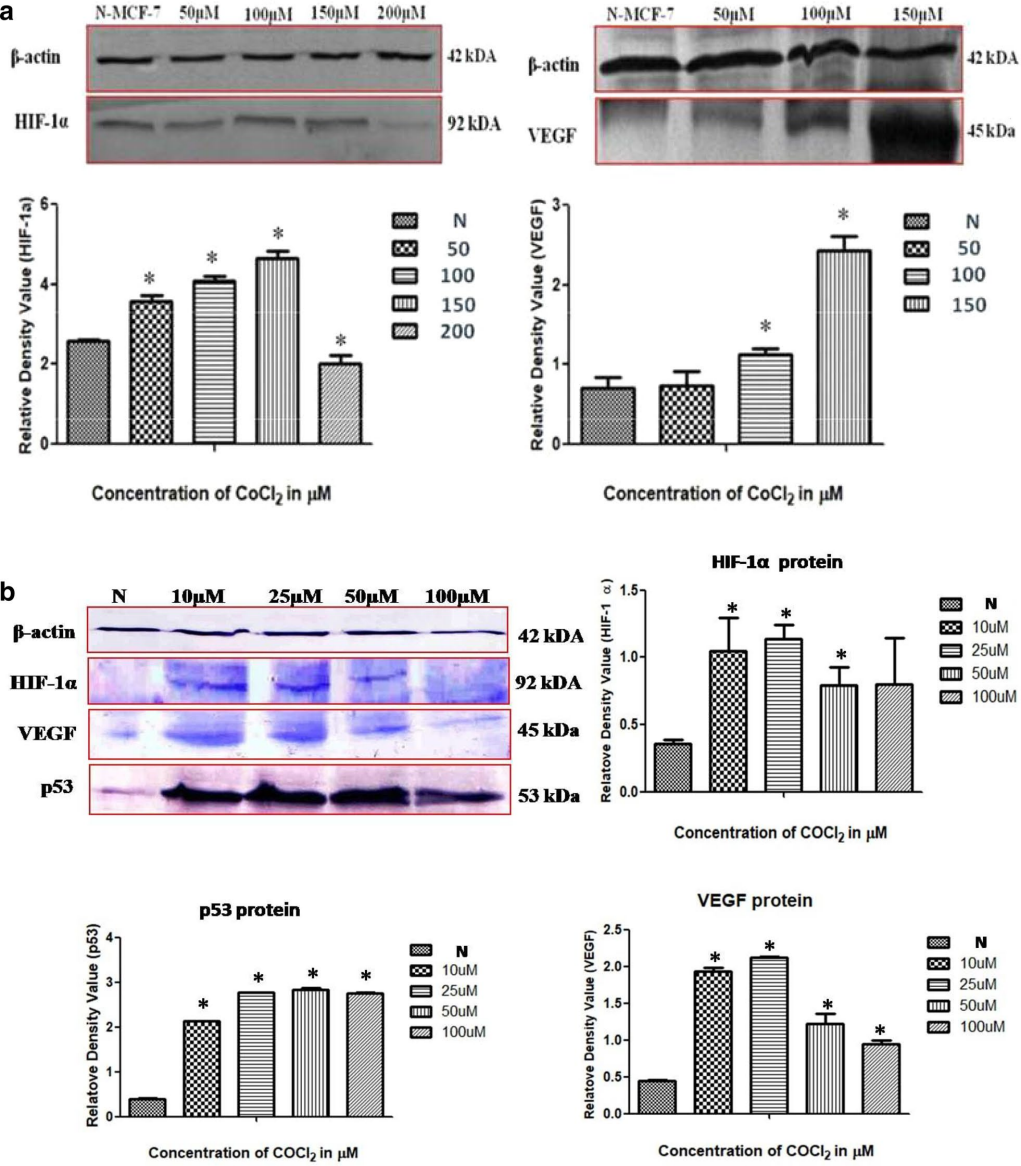
Western blot analysis of β -actin, HIF-1α, VEGF, p53 and BAX protein in breast cancer cells grown under hypoxia (treated with CoCl_2_) as well as in normoxia (without treatment). **a** Differential expression pattern of HIF-1α and VEGF protein isolated from MCF-7 cells. **b** Shows the dose dependent enhancement in expression of hypoxia associated proteins HIF-1α, VEGF, p53 and BAX in MDA-MB-231 cells grown under hypoxic condition, P < 0.05

**Fig. 6:**
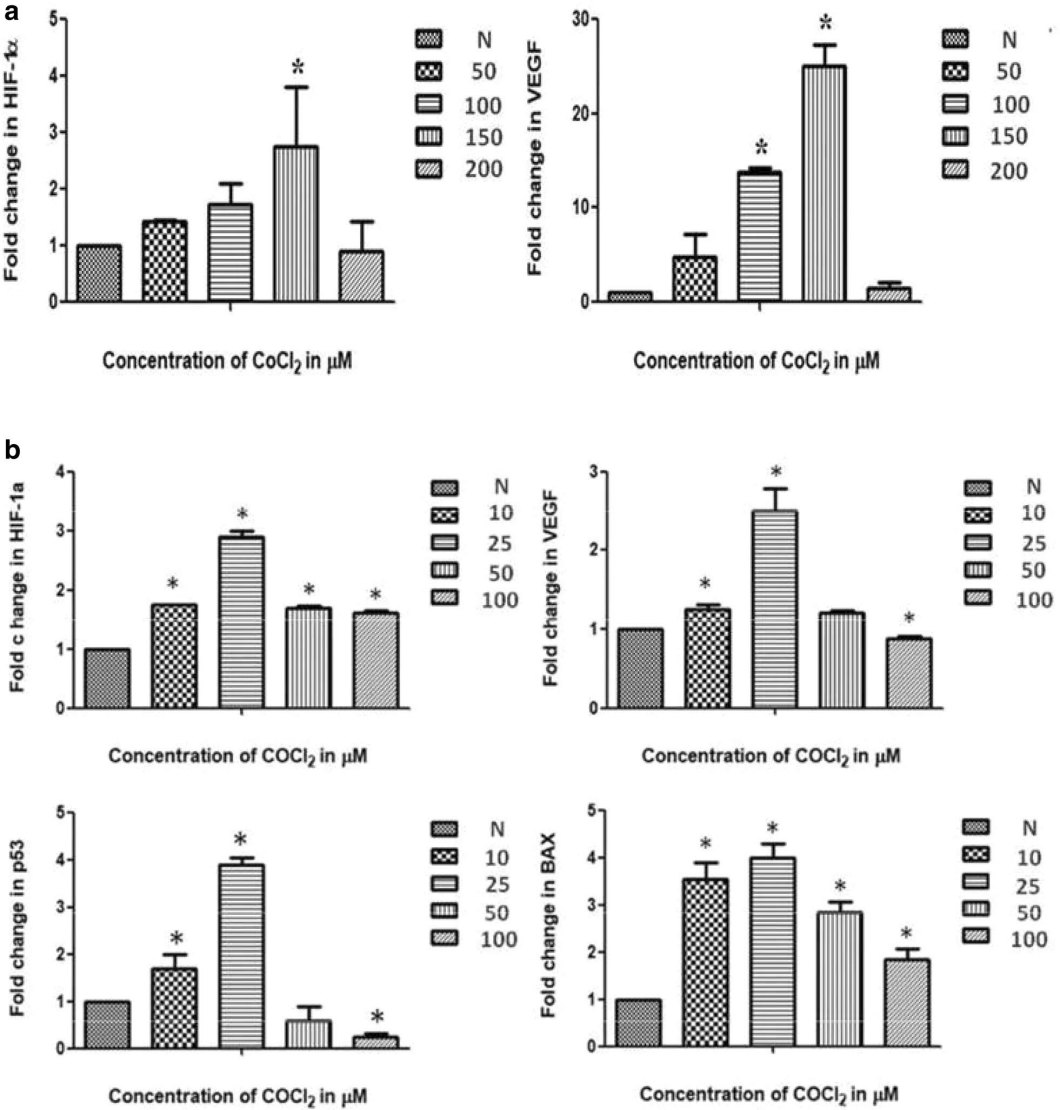
Relative fold change in expression of HIF-1α and VEGF in MCF-7 cells treated with CoCl_2_ in a dose dependent manner has been analysed in qPCR data **a** as compare to cells grown under normoxia, where β-actin used as a endogenous control. While **b** represents the qPCR data of HIF-1α, VEGF, p53 and BAX gene in MDA-MB-231 cells, P < 0.05

Validation of hypoxia induction has been done by investigating the mRNA level of HIF-1α and VEGF gene through qPCR. In addition to this mRNA level of p53 as well as BAX gene were also validated by real-time PCR in MDA-MB-231. Data from our study demonstrate a significant increase in expression level of HIF-1α (> twofold) and VEGF (> 20-fold in MCF-7 cells; > twofold in MDA-MB-231) in both the cell lines at different CoCl_2_ concentration (150 µM in MCF-7; 25 µM in MDA-MB-231) [22]. Dose dependent enhancement of HIF-1α and VEGF mRNA levels during qPCR validate the expression pattern observed at transcript as well as protein level. In addition to this dose dependent enhancement in expression of p53 as well as BAX mRNA has also been observed in MDA-MB-231. It was also found that the maximum fold change for both the genes (p53- > threefold; BAX- > threefold) were obtained at similar concentration (25 µM) which eventually validate the RT-PCR and western blot data (Figs. 4, 5, 6).

## Discussion

In the present study we first investigated whether CoCl_2_, a well known hypoxia mimetic agent [23], mediated hypoxia induction in breast cancer cells at variable concentration. Then we investigated expression pattern of hypoxia associated genes involved in angiogenesis and apoptosis during breast cancer progression under hypoxic microenvironment. Our study demonstrated that significant increase in cell proliferation up to certain concentration in both the cell lines clearly indicates the role of hypoxia in cell proliferation while the difference in concentration of CoCl_2_ at which maximum proliferation has been observed in both cells (150 µM in MCF-7; and 25 µM in MDA-MB-231) demonstrate that hypoxia induction by hypoxia mimetic agent differ with different cell types [24]. In addition to this, our study also revealed that increase in extent of hypoxia due to further increase in CoCl_2_ concentration beyond a certain level which differs for different cell types plays a crucial role in apoptotic cell death. It was reported that hypoxia show signs of dual roles in cell proliferation as well as apoptotic cell death [25]. Therefore to induce hypoxia in breast cancer cells, MCF-7 and MDA-MB-231 cells were treated with different concentration of CoCl_2_ for 72 h. Further, to investigate the effect of CoCl_2_ in cell proliferation, cell cycle analysis has been performed, a large proportion of cell population in G2/M phase were observed in both the CoCl_2_ treated cell lines as compared to untreated cells. However, further increase in CoCl_2_ concentration triggers apoptotic cell death and population of cells in G0/G1 phase increases significantly in both cells which may due to increase in extent of hypoxia by further increase in CoCl_2_ concentration that drags cells towards apoptosis [26]. Morphological study also confirms the cell proliferation that occurs in a dose dependent manner up to certain concentration in both the cell lines (MCF-7, 50–150 µM; MDA-MB-231, 10–25 µM) and further increase in CoCl_2_ concentration disturbs the cellular morphology which ultimately leads to cell death [27]. It was reported that CoCl_2_ artificially induce the hypoxia by blocking the degradation of HIF-1α [18, 19] and when HIF-1α gets activated it further stimulates transcription of hypoxia associated genes like VEGF which promotes angiogenesis [20]. In order to evaluate the hypoxia induction we first examined the expression of HIF-1α and its associated gene like VEGF as the hypoxia is well known inducer of angiogenesis which is necessary for tumor progression [28]. It was observed that CoCl_2_ induced HIF-1α accumulation and consequently up-regulated the expression of VEGF in dose dependent manner in both the cell lines. Significant increase in the level HIF-1α and VGEF confirms that CoCl_2_ artificially induce hypoxia by blocking the degradation HIF-1α which normally occurs in the presence of adequate oxygen or normoxia [22] Several reports suggest that CoCl_2_ and hypoxia regulate the expression of similar group of genes like HIF-1α, VEGF and p53 [29]. Hypoxia is well known to induce p53 expression and modulate the p53 pathway, in a HIF-1α dependent or independent manner [9]. It was also reported that accumulation of p53 protein induce apoptosis and the presence of HIF-1α and p53 seems to be important for hypoxia induced cell death [30]. Our present study indicate that there was significant increase in expression of p53 in a dose dependent manner in both the cells which confer the HIF-1α dependent induction of p53 at transcript as well as protein level, further validated by qPCR. It was reported that hypoxia induces the expression of p53 which play a major role in cells survival during hypoxic condition [11]. In contrast, there were some reports which suggest that over expression of BAX gene leads to apoptosis [12]. It was also reported that over expression of pro apoptotic gene like BAX induce apoptosis in hypoxia [31]. Various study suggest that during cancer progression amount of anti-apoptotic proteins get increased in cancer cells which makes them resistant to apoptosis during conventional therapy [32, 33]. In our study we found that there was significant increase in expression of BAX in breast cancer cells up to a certain concentration of CoCl_2_, where further increase in CoCl_2_ concentration creates high extent of hypoxia which cause the over expression of proapototic gene which ultimately induces apoptosis in breast cancer cells treated with CoCl_2_. From our present study we speculated that hypoxia alters the p53 dependent pathway in HIF-1α dependent manner thereby targeting the genes involved in p53 pathways which alters the expression of pro apoptotic genes. Our data demonstrate that hypoxic environment promotes the proliferation of breast cancer cells by altering the expression of genes involved in angiogenesis and apoptosis.

## Conclusion

In summary, we primarily aimed to established a relation between CoCl_2_ simulated cell proliferation and apoptosis in breast cancer cells under hypoxic condition. Our study revealed that CoCl_2_ mediated hypoxia induction and cell proliferation in breast cancer cells is concentration dependent and differ with different cell types. In order to evaluate the effect of CoCl_2_ in cell proliferation MTT assay followed by flow cytometry has been performed simultaneously, where a large proportion of cell population in G2/M phase were observed in both the cells at their respective concentration (150 μM in MCF-7; 25 μM in MDA-MB-231). However, further increase in CoCl_2_ concentration triggers apoptotic cell death and population of cells in G0/G1 phase increases significantly. It was observed that CoCl_2_ induced HIF-1α accumulation and consequently up-regulated the expression of VEGF up to certain concentration, which confers the hypoxia induction at their respective concentration in both the cells. Consequently increase in expression of p53 and BAX in a dose dependent manner up to certain concentration where hypoxia induced significantly confers the HIF-1α dependent induction of p53 which ultimately alters the expression of pro apoptotic gene. Here we conclude that further increase in CoCl_2_ concentration creates high extent of hypoxia which cause the over expression of proapototic gene which ultimately induces apoptosis in breast cancer cells.

## Additional file

**Additional file 1: Table S1.** Table S1 illustrates designed primer sequences used for RT-PCR, where mRNA sequnce of all five genes were obtained from the Gene database (www.ncbi.nlm.nih.gov), and primers were designed by a tool for finding specific primers, Prime3Plus (http://www.bioinformatics.nl/cgibin/primer3plus/primer3plus.cgi).
