# Supplementary material for: CoCl2 simulated hypoxia induce cell proliferation and alter the expression pattern of hypoxia associated genes involved in angiogenesis and apoptosis
Source: Biol Res. 2019 Mar 15;52:12. doi: 10.1186/s40659-019-0221-z (PMC6419504; doi:10.1186/s40659-019-0221-z)
Supplement: Supplementary file 1 — Additional file 1: Table S1. Table S1 illustrates designed primer sequences used for RT-PCR, where mRNA sequnce of all five genes were obtained from the Gene database (www.ncbi.nlm.nih.gov), and primers were designed by a tool for finding specific primers, Prime3Plus (http://www.bioinformatics.nl/cgibin/primer3plus/primer3plus.cgi). [file 40659_2019_221_MOESM1_ESM.docx]

**Additional Material**

**Title: CoCl_2_ simulated hypoxia induce cell proliferation and alter the expression pattern of hypoxia associated genes involved in angiogenesis and apoptosis.**

Nishant Kumar Rana^1^, Priya Singh^2^ and Biplob Koch*

Table S1:

| **Gene** | **Primer Sequence** | |
| --- | --- | --- |
| β-actin | Forward | 5'-ATCCACGAAACTACCTTCAA-3' |
|  | Reverse | 5'-ATCCACACGGAGTACTTGC-3' |
| HIF-1α | Forward | 5'-ACTTGGCAACCTTGGATTGG-3' |
|  | Reverse | 5'-ATCTCCGTCCCTCAACCTCT-3' |
| VEGF | Forward | 5'-TGCGGATCAAACCTCACCAA-3' |
|  | Reverse | 5'-CAGGGACGGGATTTCTTGCG-3' |
| p53 | Forward | 5'-GCCCAACAACACCAGCTCCT-3' |
|  | Reverse | 5'-CCTGGGCATCCTTGAGTTCC-3' |
| BAX | Forward | 5'-CCAAGAAGCTGAGCGAGTGT-3' |
|  | Reverse | 5'-CCGGAGGAAGTCCAATGTC-3' |
